# Shear-mediated sol-gel transition of regenerated silk allows the formation of Janus-like microgels

**DOI:** 10.1038/s41598-021-85199-1

**Published:** 2021-03-23

**Authors:** Zenon Toprakcioglu, Tuomas P. J. Knowles

**Affiliations:** 1 Centre for Misfolding Diseases, Department of Chemistry, University of Cambridge, Lensfield Road, Cambridge, CB2 1EW UK; 2 Cavendish Laboratory, Department of Physics, University of Cambridge, J J Thomson Avenue, Cambridge, CB3 0HE UK

**Keywords:** Biomaterials, Gels and hydrogels, Self-assembly, Protein aggregation

## Abstract

Microcapsules and microgels consisting of macromolecular networks have received increasing attention due to their biomedical and pharmaceutical applications. Protein microgels and in particular silk-based microcapsules have desirable properties due to their biocompatibility and lack of toxicity. Typically such structures formed through emulsion templating are spherical in geometry due to interfacial tension. However, approaches to synthesis particles with more complex and non-spherical geometries are sought due to their packing properties and cargo release characteristics. Here, we describe a droplet-microfluidic strategy for generating asymmetric tubular-like microgels from reconstituted silk fibroin; a major component of native silk. It was determined using fluorescence microscopy, that the shear stress within the microchannel promotes surface protein aggregation, resulting in the asymmetric morphology of the microgels. Moreover, the structural transition that the protein undergoes was confirmed using FTIR. Crucially, the core of the microgels remains liquid, while the surface has fully aggregated into a fibrillar network. Additionally, we show that microgel morphology could be controlled by varying the dispersed to continuous phase flow rates, while it was determined that the radius of curvature of the asymmetric microgels is correlated to the wall shear stress. By comparing the surface fluorescence intensity of the microgels as a function of radius of curvature, the effect of the shear stress on the amount of aggregation could be quantified. Finally, the potential use of these asymmetric microgels as carriers of cargo molecules is showcased. As the core of the microgel remains liquid but the shell has gelled, this approach is highly suitable for the storage of bio-active cargo molecules such as antibodies, making such a delivery system attractive in the context of biomedical and pharmaceutical applications.

## Introduction

In nature, proteins are utilised by a plethora of organisms for the formation of biomaterials necessary for a wide range of functions. As a well studied example, the glands of arthropods such as the silkworm *Bombyx mori*^1^, have the ability to induce a protein phase transition from a liquid, largely disordered random coil structure, into a solid fibre which is $\beta$-sheet heavy^2–4^. This phase transition is thought to be triggered by the shear force that the worm applies when it extrudes the protein from its gland^2,5–7^. Understanding the phase transitions involved in fiber formation^8^ is of key importance in the context of improving material fabrication and producing next-generation bioinspired materials. Native silk from *Bombyx mori*, consists mainly of two proteins; fibroin and sericin^9^. Even though extraction of fibroin from this protein mixture is quite crude, reconstituted silk fibroin (RSF) has been extensively studied in the context of forming biofilms^10^ and other biomedically related tissue engineering applications^11–13^. Moreover, due to its biocompatibility, degradability and lack of toxicity, coupled with its robustness and remarkable mechanical properties, RSF is already being used as a biomaterial in nosocomial environments^14–17^. A possible route of forming such essential functional materials can be achieved through the use of microfluidics.

Microfluidics, and in particular droplet-based approaches, have gained much interest in the area of material related applications. Materials such as hydrogels/microcapsules^18,19^ or micro-gels^20–23^ can be formed through the use of droplet-microfluidics^24^. In this platform, two immiscible phases (typically an oil phase and an aqueous phase) intersect, which results in the formation of micrometer-sized droplets. Polymer or protein molecules are encapsulated within the aqueous phase and once water-in-oil droplets are generated, the propensity of these systems to self-assemble into supra-molecular structures is utilised and microcapsules can thus be formed. Such microfluidically made materials can potentially display different and/or beneficial properties as compared to their bulk counterparts^25–28^. More recently, however, there has been an increased interest in the formation of protein-based capsules for biomedically related applications^20–22^. Since protein microgels are biodegradable, biocompatible, non-toxic and immunogenic, they represent ideal candidates for the storage and delivery of cargo molecules^9^. For this reason, microfluidically-inspired formation of silk materials is continually growing, and ranges from spinning artificial fibers^29,30^, to forming micro^31,32^ and nanogels^31–33^ but even to stabilising enzyme loaded droplets^34^.

A critical aspect of any system as a vehicle for release purposes, is monodispersity. This parameter is crucial for modulating the kinetics for molecular delivery. As microfluidics offers control over capsule morphology, generating microgels using these approaches offers advantages such as reproducibility, systematicity and scalability. However, most of these systems are utilised to form spherical micro^23^ or nanocapsules^35^, where the whole structure is solid. This leads to molecules encapsulated within the microgels having reduced diffusional movement and molecular release can only effectively occur once the whole capsule has degraded. Generating core-shell microgels, and in particular tubular structures, is thus preferential as these capsules have enhanced release capabilities due to their asymmetry, however, their production remains more challenging to achieve.

In order to address the fundamental issue of generating core-shell microcapsules with various morphologies, we have explored a droplet-microfluidic platform that enables the formation of asymmetric, tubular-like microgels which are capable of protecting and storing valuable cargo molecules. Using cryo-SEM and FTIR, we confirm that the protein silk fibroin self-assembles under shear flow within the micro-channels, resulting in only the surface of the capsule aggregating, while the core of the capsule still remains liquid. We report that by measuring the fluorescence intensity of Thioflavin T (ThT), which increases its quantum yield when it binds to $\beta$-sheet structures, and by measuring the radius of curvature, the shear stress within the microfluidic channels was correlated to the amount of surface aggregation. Finally, the potential use of the asymmetric microgels for delivery applications, such as for antibody studies, is showcased by encapsulating colloidal molecules within the asymmetric capsules and monitoring the diffusion of these particles, further corroborating that the core is still liquid even though the surface is solid. Furthermore, by altering the geometry of the microfluidic junction, janus particles were formed which can potentially be used in directed motion applications. Moreover, by generating double emulsions with the asymmetric microgels encapsulated within the hierarchical droplets, we show that control over parameters such as tubular aspect ratio or number of internal microgels, can further tailor release kinetics and thus renders such microgels as ideal candidates for storage and release of cargo molecules.

## Results and discussion

**Figure 1:**
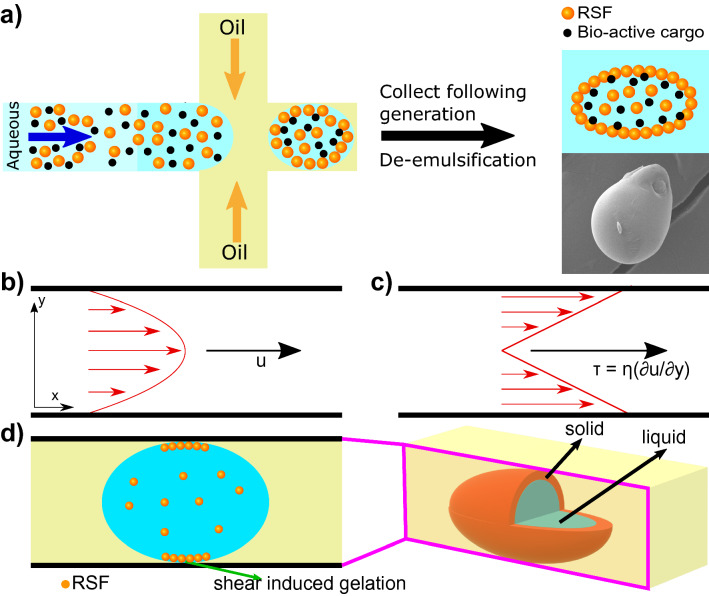
**(a)** Schematic representation of the microfluidic device used. **(b,c)** Schematic representations of the velocity profile of the fluid through a microfluidic channel (b) and the corresponding shear stress distribution (**c**). **(d)** Schematic representation of a droplet (blue) travelling within a microfluidic channel, surrounded by the oil phase (yellow) while undergoing shear. The shear stress is highest at the walls of the channel, which is where protein aggregation initiates. RSF protein is represented as orange spheres. The panel on the right is a 3-dimensional schematic of the microfluidic channel. The red area corresponds to the aggregated protein, while the blue area in the middle represents the non self-assembled and still liquid aqueous core.

The effect of shear on aggregation and on microgel morphological variations was first investigated. It is known that when protein solution travels through a microfluidic channel, it experiences shear. In fact, it has been reported that shear can induce protein aggregation^36,37^, and this effect is particularly prominent with silk-based proteins^38^. Both the spider and silkworm are capable of pulling and extruding (respectively) liquid protein from their glands and through the use of shear, a phase transition occurs which aggregates the protein into its well-known solid form. However, in the context of drug delivery, it would be particularly interesting to be able to utilise this and form capsules where the outer shell has aggregated and is solid, but internally there is still a liquid phase, i.e. a core-shell structure. To that end, and knowing that silk is shear sensitive, a high concentration of reconstituted silk fibroin (40 mg/mL) was passed through a microfluidic chip with a single junction, a schematic of which is shown in Fig. 1a, in order to generate asymmetric microgels.

It is known that the velocity profile within a channel has a parabolic curve^39^, Fig. 1b, and that the shear stress is the derivative of the velocity multiplied by the viscosity, with respect to the direction of flow movement, Fig. 1c. This is represented by the following equation, $\tau = \eta \frac{\partial (u)}{\partial (y)}$, where $\eta$ is the dynamic viscosity of the flow, u is the flow velocity of the fluid and y is the height above the boundary.

Therefore, it is clear that the shear stress is higher at the walls of the channel. When a droplet containing protein solution is pushed through such a channel, the shear force that the protein solution experiences within the droplet is highest at the walls and subsequently a reasonable assumption would be that protein aggregation would initiate there. A planar schematic representation of a droplet travelling through such a microfluidic channel is shown in Fig. 1d, where protein aggregation can be seen to initiate at the walls of the device. However, in a 3-dimensional microfluidic channel, the droplet experiences a shear force from all areas it is in contact with, and therefore, protein aggregation occurs all around the aqueous droplet. This is schematically represented in the right panel of Fig. 1d, where the red area corresponds to protein aggregates, and the blue area in the middle of the core-shell structure, is the non self-assembled, still liquid, aqueous phase.

**Figure 2:**
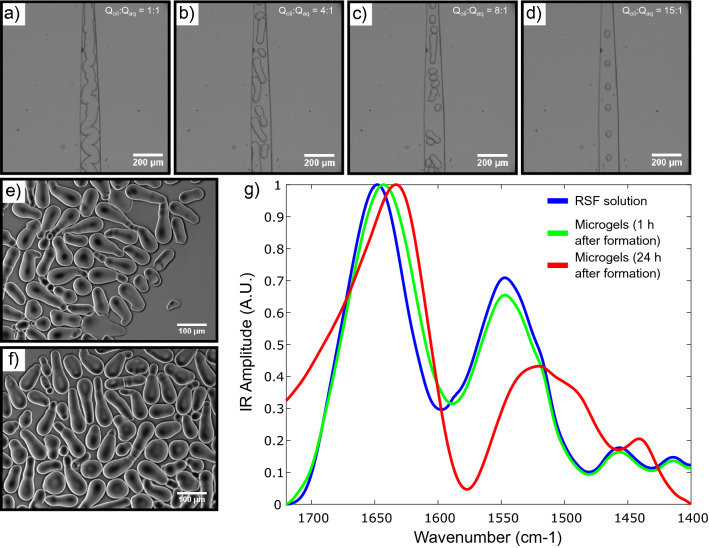
**(a–d)** Brightfield images showing the outlet of the microfluidic chip. A 40 mg/ml RSF protein was used as the dispersed phase and FC-40 with fluorosurfactant was used as the continuous phase. **(a)** Elongated fibre-like structure when $Q_{dis}$ = 100 $\mu$L/h, $Q_{cont}$ = 100 $\mu$L/h. **(b)** Asymmetric, tubular structures when $Q_{dis}$= 100 $\mu$L/h, $Q_{cont}$ = 400 $\mu$L/h. **(c)** Mixture of tubular and spherical structures when $Q_{dis}$ = 100 $\mu$L/h, $Q_{cont}$ = 800 $\mu$L/h. **(d)** spherical structures when $Q_{dis}$ = 100 $\mu$L/h, $Q_{cont}$ = 1500 $\mu$L/h. **(e)** Darkfield images of tubular structures following formation. **(f)** Darkfield images of tubular structures one week after formation. **(g)** FTIR spectra of 40 mg/ml RSF. The blue curve represents the RSF solution at t = 0 h, before any gelation has occurred. The green curve represents the micro-gels 1 h after formation, while the red curve corresponds to the micro-gels 24 h after formation.

**Figure 3:**
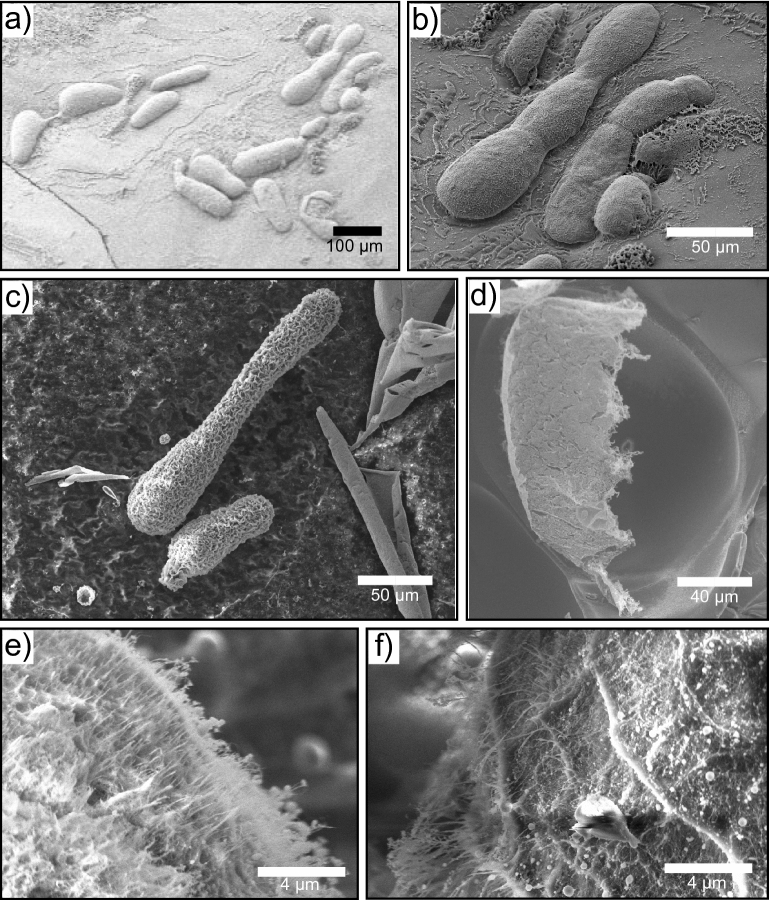
**(a–c)** Cryo-SEM micrographs of tubular/asymmetric microgels. **(d)** Micrograph of a fractured microgel which shows that the surface has aggregated whereas the core remains liquid. **(e,f)** High magnification cryo-SEM micrographs of the microgel surface. A dense fibrillar network is clearly present.

### Characterisation of the asymmetric microgels

The generation of different microgel morphologies was next investigated by varying the flow rate ratio between the aqueous and oil phases. A device consisting of height and width dimensions of 50 and $100\,\mu \hbox {m}$ respectively was used to probe microgel morphology. Initially, both the oil and aqueous phase flows were kept constant at a rate of $100\,\mu \hbox {L/h}$, which resulted in elongated structures (Fig. 2a). When the oil to aqueous phase flow rate ratio was increased to 4:1, a mixture of tubular and spherical micro-gels was formed, Fig. 2b,c, while a further increase in this ratio to 15:1 led to the generation of just spherical structures being formed, Fig. 2d. Furthermore, it appears that these structures were not only stable just after formation, but remain so for longer than 24 h, as can be seen in Fig. 2e,f. In order to determine the structural transitions undergone by the sheared silk fibroin, FTIR measurements were conducted. RSF solution was kept in the fridge at $4\,^{\circ }\hbox {C}$ prior to all experiments. The silk fibroin before gelation (blue curve) has peaks at $1650\hbox { cm}^{-1}$ and $1545\hbox { cm}^{-1}$ which are characteristic of random coil conformation and correspond to the amide I and II bands respectively. As can be seen from the spectrum in Fig. 2g, following droplet generation, the amide I peak shifted, suggesting that partial aggregation (and gelation) had occurred (green curve). Moreover, an FTIR spectrum following overnight incubation (red curve) was obtained. The amide I and II peaks had further shifted to $1630\hbox { cm}^{-1}$ and $1520\hbox { cm}^{-1}$ respectively confirming the transition from random coil to beta sheet structure. All samples were incubated at room temperature and all FTIR spectra were conducted at room temperature conditions.

In order to provide a systematic control of the RSF solution, FTIR spectra were taken for RSF solution before and after incubation at room temperature for 24 h. The peaks of the two spectra at the amide I and II bands are almost identical, indicating that the protein did not undergo any structural change (SI Fig. 2a). Moreover, the RSF solution was put in an eppendorf tube and imaged just before and after incubation at room temperature for 24 h. It is clear from these images that the protein has not gelled (SI Fig. 2b).

The surface structure of the asymmetric microgels was visualised using cryo-scanning electron microscopy (cryo-SEM). Microgels were formed and immediately de-emulsified (see Methods) before being re-immersed into an aqueous phase. The overall tubular/asymmetric morphology can be seen in Fig. 3a–c. A fracture on one of the microgels (Fig. 3d) reveals that the surface of the particle has aggregated and formed a fibrillar network, while the core of the microgel still contained protein solution. The outline of the microgel remains and can clearly be seen. Furthermore, in order to probe the surface of the asymmetric microgels in more detail, high magnification images were taken. The micrographs reveal the presence of a dense fibrillar network with fibrils pointing outwards (Fig. 3e,f).

### Effect of flow rate on shear-induced microgels and quantification of aggregates on microgel surface

The effect that flow rate, and subsequently shear rate and shear stress, have on the morphology of the formed droplets was determined and correlated to the amount of aggregates present at the surface. This was done by measuring the radius of curvature of the microgels as a function of flow rate. To correlate the flow rate with the shear stress at the walls of a rectangular channel, the following relation was used, $\tau = \eta \frac{QP\lambda }{8A^2}$, where $\eta$ is the dynamic viscosity of the flow, *Q* is the flow rate, *A* is the cross-sectional area of the microfluidic channel, *P* is the hydraulic diameter (or the wetted perimeter) and $\lambda$ is a shape factor defined by $\lambda = 24 / [(1-0.351\frac{b}{a})(1+\frac{b}{a})]^2$, where *a* represents the long side of the rectangle and *b* represents the short side^39^.

**Figure 4:**
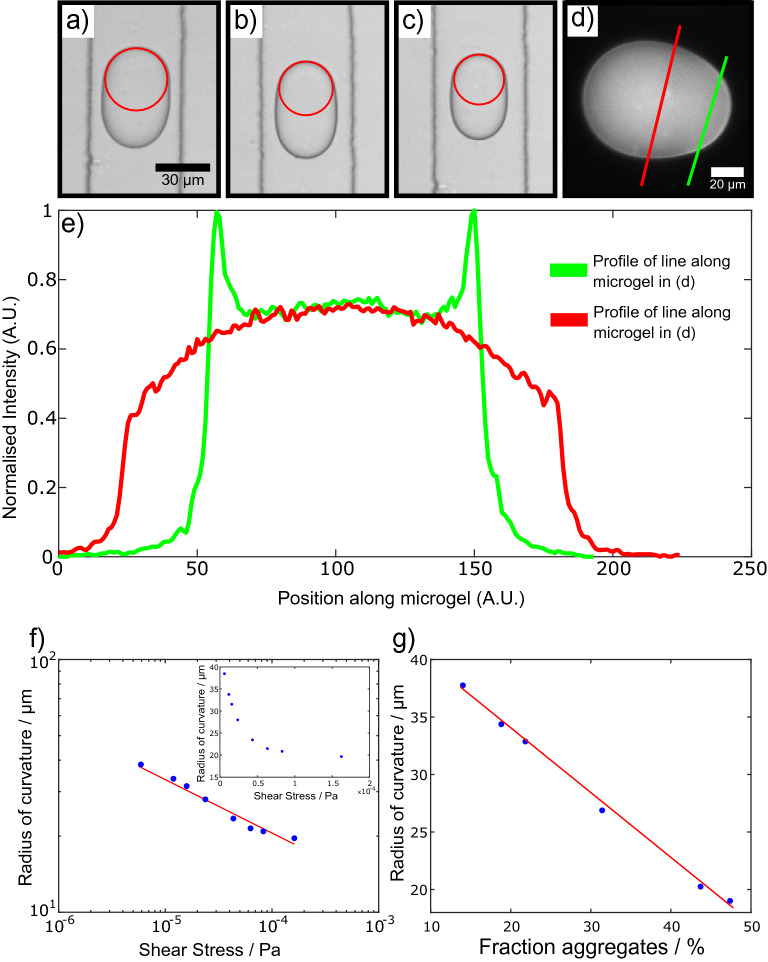
**(a–c)** Asymmetric microgel formation due to variations in the flow rate and shear stress. The dispersed phase flow rate remained constant at $100\,\mu \hbox {L/h}$, while the continuous phase flow rate was $200\,\mu \hbox {L/h}$, $300\,\mu \hbox {L/h}$ and $500\,\mu \hbox {L/h}$ respectively for each panel. The red circle indicates how the radius of curvature was determined for each system with different flow conditions. **(d)** Fluorescence micrograph of asymmetric microgels. The microgels exhibit a higher fluorescence signal at the asymmetric areas. **(e)** Graph of normalised intensity against position along the microgel for the two line profiles of the microgel in (**d**). The red curve corresponds to the line profile along the spherical part of the microgel while the green curve represents the line profile at the asymmetric part. **(f)** Double logarithmic plot of the radius of curvature against shear stress. The fit to the data is shown as a solid red line. Inset: Graph of radius of curvature against shear stress. **(g)** Graph of radius of curvature against percentage of aggregates.

Therefore, by changing the flow rate, the shear stress was systematically varied and the amount of surface aggregation could be monitored. The most stable configuration of a water in oil droplet is a sphere. In fact, if the droplets only contained an aqueous phase then the interfacial tension would promote the minimisation of the surface energy resulting in the droplets adopting a spherical shape. Therefore, any asymmetric microgel must contain elements of aggregated protein. Consequently, the radius of curvature is proportionate to the amount of aggregates present. Moreover, the more the surface has aggregated, the smaller the radius of curvature. A systematic change of the shear flow was thus conducted and the radius of curvature was measured. Examples of asymmetric microgel formation through varying flow rates are shown in Fig. 4a–c. Furthermore, a red circle around the edge of the microgel indicates how the radius of curvature at that particular area was determined for each flow rate system.

The graph of wall shear stress as a function of radius of curvature is plotted in Fig. 4f inset. It is clear that the data follow an asymptotic trend, where a high shear stress corresponds to a smaller radius of curvature, which in turn suggests a larger amount of aggregates. A double logarithmic plot of the radius of curvature against the shear stress, shown in Fig. 4f, indicates a power law with an exponent of 0.22, as obtained by the fit.

In order to further investigate how shear affects the protein solution within the droplet, a fluorescent dye was added to the aqueous solution. The fluorescent dye Thioflavin T (ThT), has the tendency to increase its quantum yield when it interacts with protein aggregates and in particular with $\beta$-sheet structures. Therefore, a higher fluorescence intensity corresponds to the presence of protein aggregates. Following droplet generation through the microfluidic chip, the microgels were collected and imaged using fluorescence microscopy. Figure 4d shows characteristic asymmetric silk-based microgels. In Fig. 4d it can be seen that the microgels have a higher fluorescence intensity at regions which are not spherical, indicating the presence of a solid, aggregated surface and the formation of a shell at that particular area. It should be noted that although ThT does preferentially bind to $\beta$-sheet structures, it also binds to non-fibrillar protein solutions^40^. Therefore, the fact that the core displays a fluorescence signal is to be expected.

Moreover, it is evident from Fig. 4d that only areas which are asymmetric exhibit a higher fluorescence signal. A comparison between the spherical and non-spherical part of the microgel was conducted. A line profile along the spherical part (red line) and along the asymmetric part (green line) was taken and is shown in Fig. 4e. From the data, which are normalised with respect to the maximum intensity, it is clear that the mean intensity value within the droplet is around 0.7. The red curve, which represents the line profile at the circular region follows a gradual intensity increase, with a maximum in the middle of the droplet, as is expected. However, the line profile along the asymmetric area (green curve) has two prominent peaks, which correspond to the aggregated part of the microgel. The relative difference between the mean intensity value within the droplet and at the surface of the asymmetric part for this particular microgel, is around 25%. Such an analysis was conducted for 10 droplets and it was found that the relative change between the asymmetric and circular areas was 27% ± 3%.

Finally, by comparing the increase in fluorescence signal of ThT as it binds to $\beta$-sheets, the amount of aggregates present at the surface were quantified for each system of microgels formed under different shear stress conditions. The graph of surface aggregates formed as a function of the radius of curvature is shown in Fig. 4g. The shear stress has thus been correlated to the amount of surface aggregates, and therefore for a given flow rate one can predict how many surface aggregates will be formed. It should be noted, that for the ThT concentration used in this study ($50\,\mu \hbox {M}$), there is an intrinsic self-fluorescence background and thus appropriate background subtractions were made in order to produce Fig. 4g.

In order to establish that shear stress rather than other effects, such as the oil interface interacting with the protein, are responsible for promoting aggregation, low shear stresses were used. By applying small flow rates ($Q_{dis}=10 \mu \hbox {L/h}$ and $Q_{cont}=50 \mu \hbox {L/h}$) and low protein concentrations (4 mg/mL), then spherical droplets are observed, indicating that these are completely liquid as expected since there is very little shear that is not enough to induce surface aggregation (SI Fig. 2a). Moreover, ThT fluorescence images shown in SI Fig. 2b have uniform intensity overall, further suggesting that the droplets are completely liquid. An additional confirmation that the droplets are completely liquid was performed by collecting the droplets and shaking slightly. Droplet coalescence was observed suggesting that the surface of the droplets had not gelled. Conversely, when the same experiment was conducted for the tubular microgels, no coalescence was seen.

### Asymmetric tubular microgels for storage and release of cargo molecules

**Figure 5:**
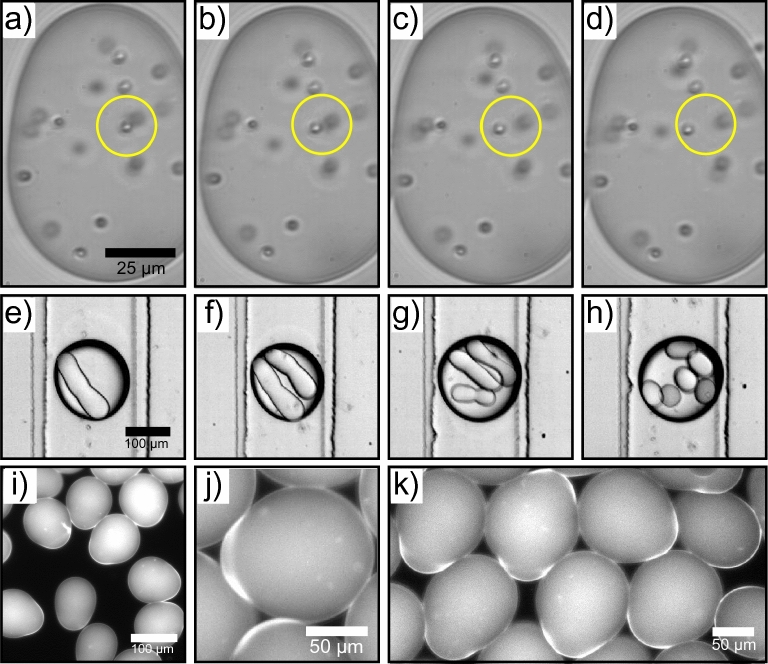
**(a–d)** Time-lapse microscopy sequence of asymmetric core-shell microgels with colloidal particles encapsulated within them. The yellow circles show an example of a colloidal pair, where the particles move with respect to each other freely within the microgel, indicating that the core is liquid and only the external surface has aggregated/gelled. **(e–h)** Wow double emulsions with asymmetric tubular microgels as the core part of the hierarchical structure. For (**e**–**g**), the outer phase flow ranged from $400-600\,\mu \hbox {L/h}$ respectively while the oil to protein phase flow rate ratio was kept constant at 4:1. For (**h**), the oil to protein phase flow rate ratio was 7:1, while the outer phase flow rate was $400\,\mu \hbox {L/h}$. **(i–k)** Fluorescence microscopy images of janus-like microgels formed using a microfluidic device with a $60^{\circ }$ junction.

Finally, the potential use of the asymmetric microgels for storage and release of cargo molecules was investigated. $5\,\mu \hbox {m}$ sized colloidal particles were added to the protein solution and following droplet generation, the colloids were encapsulated within the microgel. The tubular microgels were then collected and imaged using brightfield microscopy. A time-lapse image sequence (Fig. 5a–d and SI Videos 1–3) reveal that even though the surface of the microgels has aggregated/gelled, the core remains liquid, which is clear from the diffusion exhibited by the colloidal particles. The yellow circles in Fig. 5a–d show an example of a colloidal pair, where there is clear diffusion of these two particles with respect to each other. Such a system, where the surfaced has gelled but the core remains liquid, can be used for pharmaceutically related applications such as antibody delivery. In this approach, anything encapsulated within the core of the microgel is protected, while it is still free to diffuse, making these asymmetric capsules ideal for cell related encapsulation studies.

Moreover, an alternative route to controlling molecular diffusion for delivery related applications is through the use of double emulsions^41–43^. Water-oil-water (wow) or oil-water-oil (owo) hierarchical structures have previously been shown to be quite effective at tailoring release kinetics of encapsulated molecules^21,44,45^. To that effect, tubular microgels were encapsulated within a wow double emulsion. This was done by using two consecutive junctions, where the second junction has a non-planar (3-D) geometry^21^. At the first junction, the protein solution is intersected by the oil phase, leading to the formation of the asymmetric microgels. In the second, non-planar junction, the external aqueous phase intersects the microgel in oil resulting in the generation of the double emulsion. By varying the flow rates, tubular microgels with different aspect ratios, but also the number of asymmetric microgels encapsulated within the double emulsion, could be varied and controlled systematically. This is shown in Fig. 5e–h, where wow double emulsions with different number of encapsulated asymmetric structures as well as varying tubular aspect ratios can be seen.

Finally, by slightly changing the device geometry so that the angle at the junction is $60^{\circ }$ rather than $90^{\circ }$, janus-like microgels could be formed (Fig. 5i–k). Moreover, it can be seen that the microgels have a higher fluorescence intensity at regions which are not spherical, indicating the presence of a solid, aggregated surface and the formation of a shell at that particular area. Such structures have particular promise as “smart materials” and have significant advantages in directed motion as the two parts of the particle posses different physical/chemical properties which can be utilised. Furthermore, as the production of protein-janus particles is challenging, this microfluidic approach to generating monodisperse silk-based janus microgels can be quite advantageous.

## Conclusions

The generation of protein-based microcapsules for delivery related applications is of continuous interest. However, systematic control over microgel morphology can be challenging. Proteins such as silk, have the remarkable property of undergoing a phase transition from solution to solid under shear flow. Our results demonstrate that the shear force within a droplet-microfluidic device can result in the formation of asymmetric tubular-like microgels. Using a combination of FTIR and cryo-SEM, it was confirmed that the surface of the microgel has aggregated/gelled whereas the core still remains liquid. Furthermore, by varying the disperse and continuous phase flow rates, the shear stress could be altered and the subsequent change in microgel morphology could be measured. Additionally, it was determined that the radius of curvature has an asymptotic relation with the shear stress. Moreover, using a fluorescent dye which increases its quantum yield when it binds to $\beta$-sheet structures, we were able to quantify the amount of aggregates present at the surface, correlate that to the radius of curvature and, more importantly, to the shear stress. Finally, the potential use of the asymmetric microgels as vehicles for delivery applications was investigated. Colloidal particles were encapsulated within the tubular microgels and were imaged using a high speed camera. The colloids were able to move freely within the microgel, further corroborating that the core of the capsule remains liquid. As a final example of a way to tailor molecular diffusion for delivery applications through the introduction of an oil layer, the asymmetric microgels were encapsulated within a double emulsion. By varying the appropriate flow rates, both the tubular aspect ratio and number of encapsulated microgels could be specifically controlled. Moreover, the potential use of these microgels to form janus particles for release related applications is demonstrated. Such asymmetric protein microgels which have a hard shell but a liquid core, have the ability to protect valuable cargo molecules and thus have future applications in the pharmaceutical and biomedical fields.

## Materials and methods

### Device fabrication

In order to generate water-in-oil droplets, a soft photolithographic process was employed to fabricate the microfluidic devices used. The height and width of the device used to produce the tubular microgels were 50 and $100\,\mu \hbox {m}$ respectively. In brief, a $50\,\mu \hbox {m}$ thick photoresist layer (SU-8 3050, MicroChem) was spin-coated onto a silicon wafer and soft-baked for 15 min at $95\,^{\circ }\hbox {C}$. The photo-mask was placed onto the wafer, which in turn was exposed to UV light. This was postbaked for 5 min at $95\,^{\circ }\hbox {C}$. In order to remove excess photoresist, the master was developed in propylene glycol methyl ether acetate (PGMEA, Sigma-Aldrich). For the double emulsion experiments, a two-step photolithographic process was used^21^.

In order to fabricate microfluidic devices, a 10:1 elastomer PDMS to curing agent (SYLGARD 184, Dow Corning, Midland, MI) mixture was used. This was cured for 3 h at $65\,^{o}\hbox {C}$. The hardened PDMS was cut, peeled off the master and holes of 0.75 mm were punched into the PDMS. This was then bonded onto a glass slide by treating with a plasma bonder (Diener Electronic, Ebhausen, Germany).

### Droplet formation

neMESYS syringe pumps (Cetoni, Korbussen, Germany) were used to control the flow rates within the microfluidic channels. The dispersed phase consisted of a 40 mg/mL protein solution, and in the case of fluorescence based experiments Thioflavin T (Siga Aldrich) was added. Moreover, G5000 particles (Thermo Scientific) were used for colloidal experiments. The continuous phase was comprised of fluorinated oil (Fluorinert FC-40, Sigma Aldrich) containing 2% w/w fluorosurfactant (RAN Biotechnologies). All bright field images were acquired using a Mikotron high speed camera.

### Silk fibroin preparation and purification

Silk fibroin protein was extracted from *Bombyx mori* silk cocoons (Mindsets (UK) Limited) through the following protocol^9^. Initially, the cocoons were cut into pieces and placed in a beaker containing a solution of 0.02 M sodium carbonate. This was then boiled for 30 minutes, ensuring that the sericin that is present within the silk fibres, dissolved, while the insoluble fibroin remained in the beaker. The fibroin was then removed from the beaker, rinsed with cold water three times and left overnight to dry out.

A 9.3 M lithium bromide solution was prepared and added to the dried silk fibroin in a 1:4 ratio of silk fibroin to lithium bromide. The mixture was heated to $60\,^{\circ }\hbox {C}$ and left for 4 h, resulting in the silk fibroin dissolving in the lithium bromide. LiBr was removed from the solution by placing the mixture in a 3 kDa dialysis tube. This in turn was placed in a beaker containing ultrapure water, while the use of a large magnetic stir bar with a magnetic stir-plate was employed to ensure mixing. The water was changed a total of 6 times in 48 h.

Finally, the silk fibroin solution was removed from the dialysis tube and placed in Eppendorf tubes. These were then centrifuged at 9000 r.p.m. at $4\,^{\circ }\hbox {C}$ for 20 minutes in order to remove small impurities. The process was repeated twice and the final solution was stored at $4\,^{\circ }\hbox {C}$. All experiments were conducted within 2 weeks of extracting and purifying the silk fibroin to ensure no gelation had occurred.

### Microgel de-emulsification

Droplets were de-emulsified and separated from the continuous oil phase by applying the following protocol. A 20% 1H, 1H, 2H, 2H-perfluoro-octanol (PFO, Alfa Aesar) in FC-40 oil solution was prepared and added to the emulsion. An equal volume of deionised water was then added to the emulsion. The samples were centrifuged for 2 minutes at 1000 rpm resulting in separation of the phases, with the supernatant containing the micro-gels. The supernatant was collected, and the whole washing process was repeated an additional two times.

### Fluorescence microscopy and image analysis

The structural change of the protein solution was monitored and studied by looking at the change in intensity of ThT dye at 480 nm. A ThT concentration of 50 $\mu$M was used for all experiments. An inverted Zeiss microscope was employed to detect the fluorescence signal of the fluorophore thioflavin T. Appropriate filters that had an excitation wavelength of 440 nm and emission wavelength of 480 nm were used. All image analysis including measurements of radius of curvature and intensity were performed using ImageJ software.

### Cryo-scanning electron microscopy (cryo-SEM)

For cryo-sem, the sample was first mounted onto a mutli-pin specimen mount. This was then placed in liquid nitrogen to rapidly freeze, thus ensuring that the micro-gels remained intact. The samples were fractured using a knife and 4 nm of platinum was sputter coated onto the sample. Images were obtained using a Zeiss EVO HD15 operating at 6 kV to minimise beam damage.

### Fourier Transform Infrared Spectroscopy (FTIR)

The conformational changes of silk fibroin (both bulk and micro-gels) were performed by using an FTIR equinox 55 spectrometer (Bruker). For the bulk measurements, the samples were loaded onto the FTIR sample holder and were analysed by subtracting a water reference. Conversely, for the micro-gel measurements, an emulsion of water droplets in FC-40 was used as a reference. In all measurements conducted, a carbon dioxide atmospheric compensation was made by subtracting this from the FTIR spectra. All FTIR measurements were conducted at room temperature.

## Supplementary Information

Supplementary Video S1.

Supplementary Video S2.

Supplementary Video S3.

Supplementary Information.
