# Supplementary material for: Shear-mediated sol-gel transition of regenerated silk allows the formation of Janus-like microgels
Source: Sci Rep. 2021 Mar 23;11:6673. doi: 10.1038/s41598-021-85199-1 (PMC7988050; doi:10.1038/s41598-021-85199-1)
Supplement: Supplementary file 4 — Supplementary Information. [file 41598_2021_85199_MOESM4_ESM.pdf]

Shear-mediated sol-gel transition of regenerated silk allows the formation of Janus-like microgels

Zenon Toprakcioglu[†] and Tuomas P. J. Knowles[*,†,‡]

[†]*Centre for Misfolding Diseases, Department of Chemistry, University of Cambridge, Lensfield Road, Cambridge CB2 1EW, UK*

[‡]*Cavendish Laboratory, Department of Physics, University of Cambridge, J J Thomson Avenue, Cambridge CB3 0HE, UK*

E-mail: tpjk2@cam.ac.uk

Supplementary Information

SI videos 1-3: Time-lapse videos showing asymmetric core-shell microgels with colloidal particles encapsulated within them.

Acknowledgments

The research leading to these results has received funding from the European Research Council under the European Union's Seventh Framework Programme (FP7/2007-2013) through the ERC grant PhysProt (agreement n° 337969). We are grateful for financial support from the BBSRC (TPJK), the Newman Foundation (TPJK), the Wellcome Trust (TPJK) and the Cambridge Centre for Misfolding Diseases.

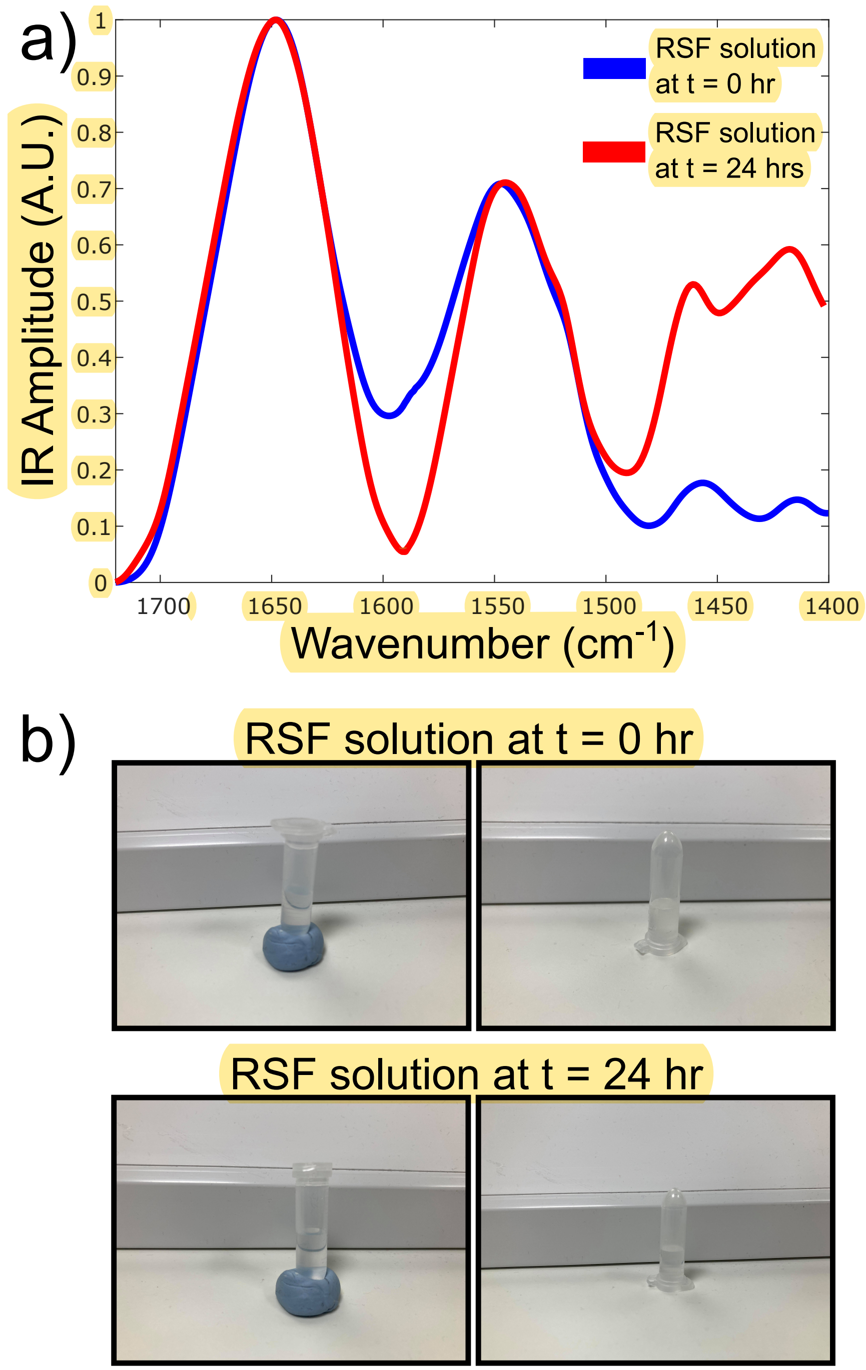

Figure 1: (a) FTIR spectra of 40 mg/ml RSF solution. The blue curve represents the RSF solution at t = 0 hr, while the red curve represents the micro-gels 24 hr after formation. **(b)** Images of the corresponding RSF solution at t = 0 hr and after 24 hr. For both measurements in (a) and (b), the solution was incubated at room temperature for 24 hr.

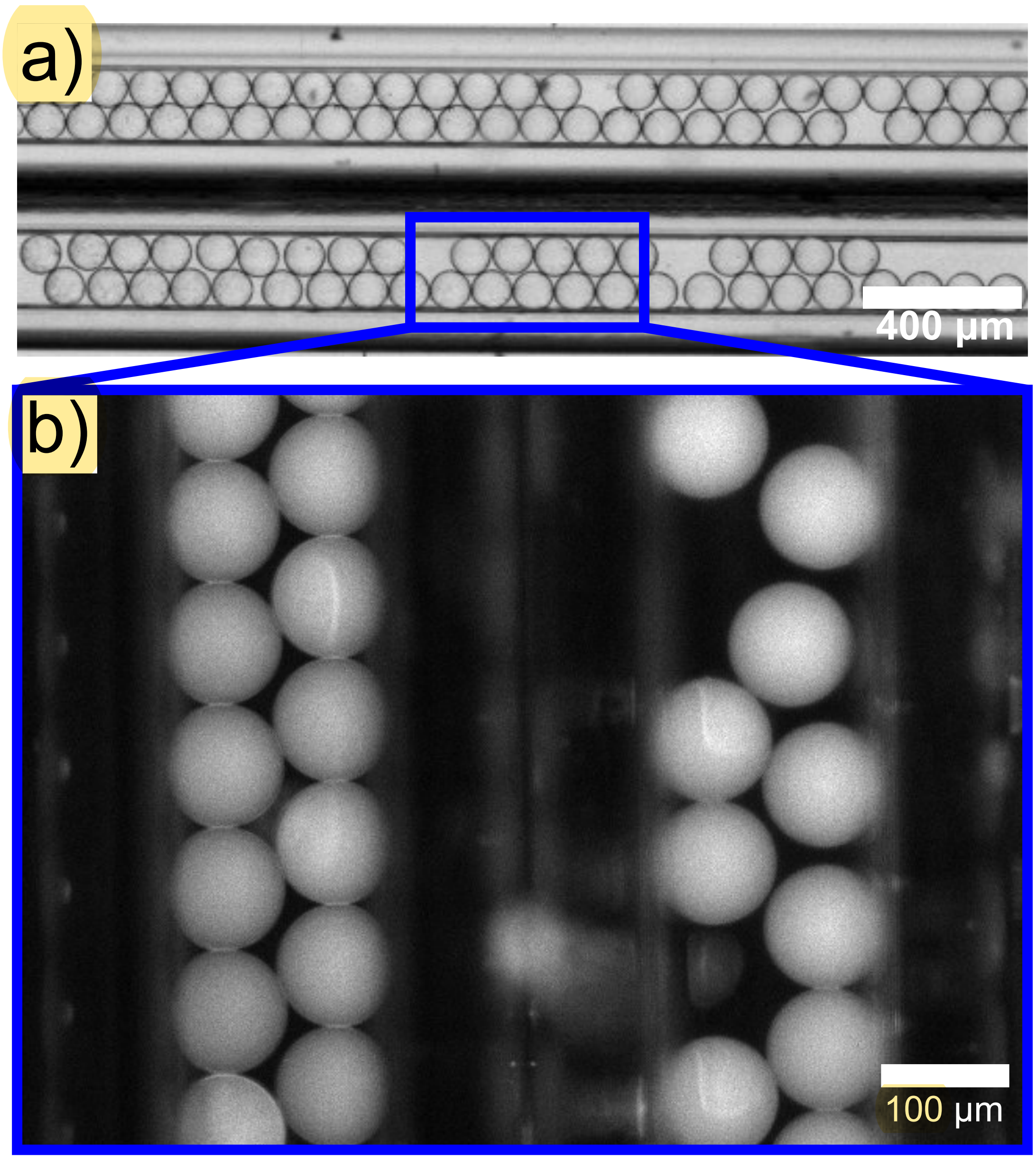

Figure 2: (a) Brightfield microscopy image of droplets consisting of silk fibroin solution, collected in a capillary following their generation. The droplets were formed using low flow rates (Q_{dis}=10 μL/hr, Q_{cont}=50 μL/hr). **(b)** Fluorescence microscopy images of the corresponding droplets. The droplets exhibit the same overall intensity, indicating that there is no surface aggregation present.
